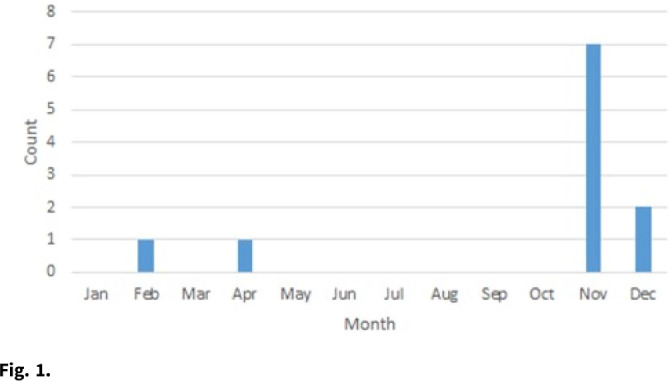# Learnings from a Cutibacterium acnes pseudo-outbreak in pediatric neurosurgical patients

**DOI:** 10.1017/ash.2022.166

**Published:** 2022-05-16

**Authors:** Felicia Scaggs Huang, Andrea Ankrum, Cincinnati Hospital, Zheyi Teoh, Joshua Courter, Francesco, Mangano, Karin Bierbrauer, Josh

## Abstract

**Background:** *Cutibacterium acnes* is normal skin flora as well as a common culture contaminant. It can cause infections in the setting of sterile implants, although clinical presentations can be subtle. Differentiating true infection from sample contamination is challenging and has implications for patient care. We describe an investigation of a cluster of 7 hospitalized pediatric patients with *C. acnes* isolated from anaerobic cultures of cerebrospinal fluid (CSF) over 3 weeks at a quaternary-care children’s hospital. **Methods:** An outbreak response was coordinated between the infection prevention and control (IPC), microbiology, and neurosurgery teams. We defined a case as a hospitalized patient with *C. acnes* isolated from a CSF culture beginning in November 2020. We reviewed charts of all cases and CSF culture collection on all case units, transport to and processing at the microbiology laboratory, and the IPC team measured adherence for all processes. **Results:** There were 8 positive cultures in 7 cases from November 10 to 27, 2020. The median case age was 2 months (range, 0–119). Cases occurred on 4 different units. All positive patients had at least 1 implanted neurosurgical device used for CSF drainage. There were no clear commonalities in surgeon responsible for device placement, hardware type placed, or staff collecting CSF samples. A standard protocol for CSF collection was followed for all cases. Overall, 3 patients cleared cultures without intervention, 2 received oral antibiotics, and 2 underwent surgical removal of their device. Specimen processing was unchanged, although due to supply issues, an alternative anaerobic culture media (Anaerobic Systems, Morgan Hills, CA) was used for 6 weeks, during which all cases were identified. Compared to routine media, the alternative is known to enhance organism detection. The company reported no concerns for media contamination or *C. acnes* outbreaks. Once routine media became available, CSF culture positivity for *C. acnes* returned to baseline (late November or early December) (Fig. 1). **Conclusions:** We identified a likely pseudo-outbreak related to temporary use of a more sensitive culture media. No direct patient harm was identified, although many had increased risk of harm by surgical intervention or prolonged length of stay. Technological advances may enhance organism identification but challenge existing paradigms of care. More studies are needed to better delineate the intersection of diagnostic advancements with patient care standards.

**Funding:** None

**Disclosures:** None

**Figure:**